# A Novel Herbal Nano-Based Ear Drop with *Ocimum gratissimum* Essential Oil: An Alternative Strategy for Managing Otomycosis

**DOI:** 10.3390/pharmaceutics18060751

**Published:** 2026-06-18

**Authors:** Bac V. G. Nguyen, Hoai Thu Le, Tien-Trung Dao, Quy-Nguyen Doan, Duc-Huy Pham, Nghi Bao Nguyen, Minh-Tri Le, Du-Thien Nguyen, Phuoc-Vinh Nguyen

**Affiliations:** 1 School of Pharmacy, University of Medicine and Pharmacy at Ho Chi Minh City, Ho Chi Minh City 700000, Vietnam; nguyenvugiangbac@ump.edu.vn (B.V.G.N.); pdhuy.d18@ump.edu.vn (D.-H.P.); 2 Center for Molecular Biomedicine, University of Medicine and Pharmacy at Ho Chi Minh City, Ho Chi Minh City 700000, Vietnam; lethuhoai@ump.edu.vn (H.T.L.); nbnghi@ump.edu.vn (N.B.N.); 3 Faculty of Pharmacy, University of Health Sciences, Vietnam National University Ho Chi Minh City, Ho Chi Minh City 700000, Vietnam; dttrung@uhsvnu.edu.vn (T.-T.D.); dnquy.d2022@uhsvnu.edu.vn (Q.-N.D.); lmtri@uhsvnu.edu.vn (M.-T.L.); ndthien@uhsvnu.edu.vn (D.-T.N.); 4 Research Center of Discovery and Development of Healthcare Products, Vietnam National University Ho Chi Minh City, Ho Chi Minh City 700000, Vietnam

**Keywords:** *Ocimum gratissimum* (L.) essential oil, acetic acid, eardrop, microemulsion

## Abstract

**Background/Objectives**: Otomycosis is a recurrent fungal infection of the external auditory canal. This disease is difficult to manage with current antifungal agents due to irritation, ototoxicity risk, and emerging resistance. Natural essential oils have been proposed as alternatives, yet their clinical application in otic formulations remains limited due to their poor solubility and stability. In this study, we report the first ear-drop formulation combining microemulsified *Ocimum gratissimum* essential oil and acetic acid for otomycosis treatment. **Methods**: The essential oil was quality-validated with eugenol content superior to 60%. A systematic formulation study was performed, and the Tween 20/isopropanol (4:1, *w*/*w*) mixture was selected as the optimal surfactant system, yielding a stable microemulsion with high encapsulation efficiency (~98%) and relevant physicochemical stability (up to 28 days). The final formulation containing 1% acetic acid and 0.3% micro-emulsified essential oil met pharmacopeial requirements in terms of appearance, pH, viscosity, and microbial limits. **Results**: Importantly, this micro-emulsified eardrop demonstrated significantly greater in vitro antifungal activity than 3% boric acid and 2% acetic acid eardrops in twelve clinical fungal isolates from Vietnamese swimmers, especially on *Curvularia*, *Cunninghamella*, *Aspergillus terreus*, and *Bipolaris*. Although less pronounced than 1% clotrimazole, the finalized formulation demonstrates better antifungal kinetics and a broader activity spectrum. **Conclusions**: This work provides relevant experimental evidence on the use of *Ocimum gratissimum* essential oil in a microemulsion delivery system and demonstrates its efficacy against clinically relevant otomycosis pathogens. The results establish a foundation for future in vivo and clinical studies.

## 1. Introduction

Otomycosis, or fungal otitis externa, is a superficial fungal infection of the external auditory canal. This disease is typically prevalent in tropical and subtropical regions due to their high humidity and temperature [1,2]. The disease accounts for 9–30% of all otitis externa cases, affecting individuals across all age groups, especially swimmers, hearing-aid users, and those with compromised local immunity [3]. Although bacterial pathogens are often implicated in ear infections, fungi such as *Aspergillus niger*, *Aspergillus fumigatus*, and *Candida albicans* are predominant causative agents of otomycosis [4,5].

Clinically, the condition manifests with pruritus, otalgia, ear fullness, flaky discharge, and transient hearing loss [6,7]. While generally not life-threatening, otomycosis causes considerable discomfort, frequent recurrence, and a marked reduction in quality of life [8]. In terms of treatment, current regimens mainly rely on topical antifungal therapy with a maximized local drug concentration [9]. Among conventional antifungal agents, the solution of 3% boric acid (in 90% ethanol) is one of the most frequently used, with an efficacy between 70% and 80% of cases [10,11]. Nevertheless, its irritation and potential ototoxicity remain challenging for clinical applications [8,12,13]. To tackle this issue, acetic acid emerges as a potential candidate with positive antifungal activities in otomycosis and minimized side effects [14,15].

In addition, in recent years, increasing antifungal resistance and the lack of novel drugs have drawn the attention of the scientific community to natural bioactive compounds. Especially, essential oils appear to be the most promising candidate with diverse pharmacological properties, including antimicrobial, antifungal, and anti-inflammatory activities [16,17,18,19].

*Ocimum gratissimum* L. (commonly known as white basil or “Hương nhu trắng” in Vietnamese) is a perennial herb, widely distributed in tropical regions. In Vietnamese traditional medicine, it has been used in the treatment of respiratory infections, gastrointestinal disorders, and skin diseases [16,17,19]. Phytochemical studies have identified eugenol, thymol, and γ-terpinene as its major constituents, conferring strong antimicrobial and antioxidant properties [20,21,22].

A study by Nweze et al. (2009) demonstrated that *O. gratissimum* leaf extract exhibited broad-spectrum antimicrobial activity against both Gram-positive and Gram-negative bacteria, as well as *Candida albicans* [23]. Similarly, Lima et al. (1993) and Silva et al. (2005) reported potent antifungal effects of *O. gratissimum* essential oil against dermatophytes, with inhibition zones exceeding 10 mm and complete fungal growth inhibition at concentrations as low as 125 µg/mL [18,19].

Despite these findings, there remains a lack of data on the antifungal activity of *O. gratissimum* essential oil against otomycosis-related fungi such as *Aspergillus* spp. and *Candida albicans*, and its potential in otic pharmaceutical formulations has not been investigated.

In the current study, we formulated and evaluated an ear drop preparation combining acetic acid and micro-emulsified *O. gratissimum* essential oil as a natural alternative to synthetic antifungal agents for otomycosis management. The formulation was characterized for its physicochemical properties and tested in vitro against clinical fungal isolates from Vietnamese swimmers.

## 2. Materials and Methods

### 2.1. Materials

*Ocimum gratissimum* essential oil (WBEO) was procured from Saroma company (Ho Chi Minh City, Vietnam), and the eugenol analytical standard was provided by Sigma-Aldrich (St. Louis, MO, USA).

Glacial acetic acid was purchased from Merck (Rahway, NJ, USA). Polyvinyl Pyrrolidone K30 (PVP K30) was provided by BASF Advanced Chemicals Co., Ltd. (Shanghai, China), and Tween 20 was supplied by Xilong Scientific (Guangzhou, China). All ingredients complied with specifications for pharmaceutical uses.

The reference drugs used in this study were 3% (*w*/*v*) Boric acid, purchased from F.T Pharma-Vietnam (Lot No. 000323, Ho Chi Minh City, Vietnam); Mepatyl^®^, provided by Merap-Vietnam (Lot No. 0040622, Ho Chi Minh City, Vietnam), and Candid^®^, purchased from Glenmark-India (Lot No. 10230769, Mumbai, India).

#### 2.1.1. Clinical Strains

##### Sample Collection and Ethical Approval

Twelve fungal strains used in this study were isolated from Vietnamese swimmers diagnosed with otomycosis. The research protocol was reviewed and approved by the Biomedical Research Ethics Committee of Pham Ngoc Thach University of Medicine (Approval No. 441/HĐĐĐ/TĐHYKPNT, dated 11 December 2020). The participants ranged in age from 5 to 25 years, with the majority belonging to the 8–12 and 19–21 age groups. The strains were identified by ITS sequencing. Each collection tube was prepared by adding 2 mL of 0.85% sterile saline containing 0.05% Tween 80 and a sterile cotton swab to a screw-cap test tube, followed by autoclaving. Dr Ha Nam Nguyen (MD, PhD), an otolaryngologist from Pham Ngoc Thach University of Medicine, conducted otoscopic examinations and recorded relevant demographic and clinical information. Specimens were then collected by swabbing the external auditory canal using sterile cotton swabs moistened with saline solution. Following the collection, samples were transported under aseptic conditions and processed for fungal isolation within one hour in a Class II biosafety cabinet. The isolates were subsequently stored at −80 °C until further microbiological and antifungal testing.

### 2.2. Methods

#### 2.2.1. Quality Control of *O. gratissimum* Essential Oil

The essential oil of *O. gratissimum* used in this study was evaluated for quality compliance according to the Vietnamese Pharmacopeia V standards [24], including the following:

Organoleptic properties: The oil was required to be a clear, pale-yellow liquid with a characteristic aromatic odor and a spicy, warm, and slightly numbing taste.

Qualitative: The identity of the oil was confirmed by high-performance liquid chromatography (HPLC) equipped with a photodiode array (PDA) detector. The chromatographic profile of the sample exhibited a major peak corresponding to the retention time of the eugenol reference standard.

Quantitative: Quantitative analysis of eugenol content was performed by HPLC–PDA. The essential oil met the pharmacopeia specification, with eugenol concentration exceeding 60% of the total composition.

#### 2.2.2. Eardrop Formulation Containing *O. gratissimum* Essential Oil and Acetic Acid

In our previous study, we investigated the minimum inhibitory concentrations (MIC) of *O. gratissimum* essential oil and acetic acid, as well as the optimal mass ratio of the two active components, determined to be 0.3:1.0 (respectively) [25]. Therefore, this ratio was adopted in the formulation development of the present study.

#### 2.2.3. Screening of Surfactant/Co-Surfactant

The microemulsion formulation was developed using a combination of surfactant and cosurfactant. The screening of surfactants and co-surfactants was conducted based on their solubilization capacity for WBEO. Briefly, binary mixtures were prepared by incorporating each candidate surfactant/co-surfactant at a 1:1 (*w*/*w*) ratio. The mixtures underwent high-speed vortex homogenization for 24 h to ensure maximal interfacial contact. The resulting mixtures were assessed visually for isotropy and phase separation. Only systems yielding a homogenous, transparent phase were selected for further studies. In total, four surfactants (Tween 20, Tween 80, Span 20, and Cremophor RH40) and four co-surfactants (ethanol, glycerol, propylene glycol, and isopropanol) were assessed.

#### 2.2.4. Selection of Surfactant-to-Co-Surfactant Ratios

Following the identification of a suitable surfactant/co-surfactant, the influence of the surfactant-to-co-surfactant mass ratio was evaluated. These mixtures (S_mix_) were systematically varied (2:1, 3:1, and 4:1 *w*/*w*). To assess the emulsification efficiency and evaluate the phase behavior, 0.10 g of WBEO was blended with each Smix at a fixed 1:1 (*w*/*w*) ratio. This mixture was subjected to aqueous titration with 0.10 mL increments of distilled water under continuous agitation at ambient temperature. The titration proceeded until either a total weight of 20 g was attained or phase turbidity was observed. To ensure thermodynamic equilibrium and verify the kinetic stability of the resulting systems, all transparent formulations were maintained under identical stirring conditions for an additional 24 h. Only those samples that retained monophasic isotropy without evidence of phase separation after this period were selected for further optimization.

#### 2.2.5. Construction of Pseudo-Ternary Phase Diagrams

Based on the screening, the selected surfactant/co-surfactants were further investigated for their emulsification efficiency by varying the Smix ratio to WBEO. Pseudo-ternary phase diagrams were constructed using the water titration method at ambient temperature (25 °C). The oil phase (0.50 g of WBEO) and the Smix were blended at precise weight ratios ranging from 1:0.5 to 1:10 (*w*/*w*). These mixtures were titrated with distilled water under moderate magnetic stirring until either a total weight of 10 g was attained or the onset of turbidity was observed, marking the phase transition from an isotropic microemulsion to a biphasic system.

The resulting coordinate points were mapped onto ternary plots to identify the microemulsion domain. Selection of the final formulation was based on three specific physicochemical constraints: a maximal microemulsion area that accommodated a surfactant/co-surfactant concentration below 25% (*w*/*w*) while maintaining high aqueous loading (water content ≥ 50% *w*/*w*). These criteria were established to ensure both the dermatological safety of the otic delivery system and the thermodynamic stability of the internal phase across a broad concentration gradient, ranging from a minimum loading of 5% (*w*/*w*) to a maximum threshold of 15% (*w*/*w*).

#### 2.2.6. Physicochemical Characterization of the Microemulsion

To evaluate the colloidal stability of the selected microemulsion, it was monitored over a sequential 28-day period at ambient temperature (25 °C). The evaluation was conducted in two distinct phases, with morphological characteristics re-assessed at 1-week and 2-week intervals, both before and after acidification.

- Phase I: Characterization was performed at T0 (day of compounding), followed by T7 and T14. This phase established the fundamental stability of the system interface under neutral conditions.
- Phase II: Following the incorporation of 1% (*w*/*w*) acetic acid at the 14-day mark, the system was reassessed at T21 and T28. These intervals were utilized to evaluate the pH-resilience of the nanostructure and ensure that the electrolyte addition did not compromise the microemulsion architecture.

#### 2.2.7. Visual Assessment

The microemulsions must exhibit sustained optical isotropy and persistent phase homogeneity. Any manifestation of macroscopic phase separation, creaming, or solute precipitation, either before or after the 1% acetic acid incorporation, will result in the rejection of the formulation candidate.

#### 2.2.8. Droplet Size, Polydispersity, and Surface Charge

The mean hydrodynamic diameter (D_H_), polydispersity index (PDI), and zeta potential were determined using dynamic light scattering (DLS) with a Zetasizer Nano ZS90 (Malvern Instruments, Malvern, UK). Measurements were performed in triplicate at ambient temperature. To minimize multiple scattering effects and ensure an optimal count rate, formulations were appropriately diluted with deionized water prior to analysis.

### 2.3. TEM Analysis

For TEM sample preparation, the microemulsion was first ultrasonicated for 5 min to obtain a homogeneous dispersion. Subsequently, 5–10 μL of the microemulsion was drop-cast onto a carbon-coated copper grid (300 mesh) and allowed to dry naturally at room temperature for 2 h. Transmission electron microscopy (TEM) analyses were performed using a JEOL JEM-F200 (HRP) microscope (JEOL, Tokyo, Japan) operated at an accelerating voltage of 200 kV.

#### 2.3.1. pH Measurement

The apparent pH of the microemulsions was measured using a calibrated digital pH meter (S220-Kit, Mettler Toledo, Seoul, Republic of Korea) at ambient temperature.

#### 2.3.2. Phase Stability

The phase stability of the obtained microemulsion was assessed using centrifugation and thermal stability methods, as follows:

*Accelerated physical stability via centrifugation:* To assess kinetic stability against gravitational separation, the microemulsions were subjected to centrifugation at 4000 rpm for 30 min at ambient temperature. Formulations were monitored for coalescence, creaming, or phase stratification; only systems maintaining visual isotropy and transparency were deemed stable.

*Thermal stability:* Thermal stability was evaluated using an accelerated stress protocol. Formulations were subjected to a cyclic temperature challenge of 2–8 °C, ambient temperature, and 50 °C for 24 h at each interval. This sequence was repeated for six consecutive cycles (*n* = 6). The integrity of the nanostructures was verified by the absence of turbidity or phase separation.

#### 2.3.3. Encapsulation Efficiency

The encapsulation efficiency (EE%) was determined indirectly by quantifying the non-encapsulated (free) eugenol. A 1.5 mL aliquot of the microemulsion was centrifuged at 12,000 rpm for 10 min to separate the aqueous phase. The supernatant (0.5 mL) was diluted in methanol to a 10 mL final volume and filtered through a 0.22 µm membrane. Eugenol concentration was quantified using HPLC. The EE% was calculated using the following equation:

$$\mathrm{EE}\%=\frac{C_{\mathrm{total}}-C_{\mathrm{free}}}{C_{\mathrm{total}}}$$

wherein C_total_ represents the initial concentration of eugenol added, and C_free_ is the concentration detected in the supernatant. All analyses were performed in triplicate (*n* = 3).

#### 2.3.4. Antifungal Activity Assessment

Building upon these selections, a series of otic formulations was engineered comprising bioactive agents (WBEO and acetic acid), a mixture between surfactant and co-surfactant, and PVPK30 1% (*w*/*w*) as thickener. Based on our preliminary optimization studies, which established an optimal synergistic mass ratio of 0.3:1 (WBEO to acetic acid), the essential oil concentration was subsequently designated as 0.3% (*w*/*w*). To evaluate the influence of the nanocarrier system on bioactivity, three distinct formulations were prepared for antifungal ability assessment (Table 1):

- Formulation 1 (Control I): WBEO was solubilized directly in the solution of PVPK30 1% in dimethyl sulfoxide (DMSO) to serve as a bulk oil control.
- Formulation 2 (Control II): WBEO and acetic acid were sequentially dissolved in the solution of PVPK30 1% in DMSO to assess the baseline synergistic effect of the active ingredients in a conventional solvent system.
- Formulation 3 (Eardrop containing microemulsified WBEO): A lipid-surfactant premix was generated by incorporating the WBEO into the Smix under continuous magnetic stirring, followed by the integration of acetic acid, and PVPK30 1%.

For all preparations, the final weight was adjusted to 100 g using DMSO (for Controls) or distilled water (for the microemulsion). The resulting systems were stored in LDPE containers under light-protected conditions for subsequent physicochemical and biological characterization. In addition, three commercialized products for otomycosis treatment, including boric acid 3%, acetic acid 2% (Mepatyl^®^), and clotrimazole 1% (Candid^®^), were used as positive control.

For the antifungal activity testing procedure, 2 mL of sterile saline solution (0.85% NaCl) containing 0.5% Tween 80 was added to a fungal slant culture. The spores were gently dislodged and suspended to obtain a homogeneous spore suspension, which was then transferred to a sterile tube. The optical density (OD) of the suspension was measured at 530 nm and adjusted to approximately 0.08–0.10, corresponding to a concentration of 1–5 × 10^6^ CFU/mL.

The antifungal activity was evaluated using a modified agar well diffusion method as described by Balouiri et al. [26]. Briefly, 20 mL of Potato Dextrose Agar (PDA) was poured into each sterile Petri dish and allowed to solidify. The agar surface was then evenly inoculated using a sterile cotton swab previously immersed in the standardized fungal suspension. Wells of 6 mm in diameter were aseptically created in the agar using a sterile cork borer, and 100 µL of the test sample was added to each well.

The inoculated plates were incubated at room temperature for three days. Following incubation, the diameters of the inhibition zones were measured using digital calipers. All experiments were conducted in triplicate, and the mean inhibition zone diameters were measured.

#### 2.3.5. Antifungal: Kinetic Activity Assessment

A volume of 900 µL of fungal spores was subsequently exposed to 100 µL of the test preparations. A parallel control group, consisting of fungi in physiological saline, was included for comparison. The number of viable fungi was then quantified at exposure intervals of 10, 30, and 60 min.

The antifungal efficacy of the preparations was determined by calculating the percentage of fungi eliminated. The fungal killing kinetics over time were plotted using GraphPad^®^ software (version 9.5.0.730). All experiments were tripled.

#### 2.3.6. Quality Specifications for the Finalized Formulation

Three different batches of the finalized formulation (100 units per batch (10 g per unit)) were fabricated. For each batch, six samples were randomly taken and analyzed, as follows:

Appearance: Pale yellow solution.

pH: Dilute the preparation with distilled water at a 1:1 ratio. Measure the pH of the diluted preparation using a pH meter with a calomel-glass electrode pair. The test volume should be approximately 10 mL. Perform three measurements at room temperature for each sample. The pH of the eardrop formulation should be between 2.0 and 4.0.

Viscosity: Measure using a Brookfield Ametek DV1 viscometer (AMETEK Brookfield, Middleboro, MA, USA) at room temperature, with a rotation speed of 30 rpm and a sample volume of 0.5 mL. Perform three measurements. The viscosity of the formulation should be between 14.9 and 18.3 cP.

Microbial limits: The total aerobic microbial count should not exceed 10^2^ CFU/mL, and the total fungal count should not exceed 10^1^ CFU/mL. The sample should be free of *Pseudomonas aeruginosa* and *Staphylococcus aureus* in 1 g of the preparation.

Identification

*O. gratissimum* essential oil: The retention time of the eugenol peak in the chromatogram of the sample solution with the HPLC-PDA method must correspond to the retention time in the chromatogram of the eugenol standard.

Acetic acid: Dissolve 5 mL of preparation with 10 mL of distilled water, then adjust to pH 7 with 1N NaOH. Then add 9% FeCl_3_ solution. A brick red precipitate is formed. Heat the preparation with concentrated sulfuric acid and absolute ethanol. The product of the reaction has a characteristic odor of ethyl acetate. The formulation must possess characteristic reactions of the acid group and the acetate group.

Assay

Eugenol content: HPLC-PDA method was used as described in Section 2.3.4. The eugenol content in the solution should be between 90.0% and 110.0% of the labeled amount.

Acetic acid content: The acid-base titration method was used. The acetic acid content in the solution should be between 85.0% and 130.0% of the labeled amount.

### 2.4. Cytotoxicity Assay

The cytotoxicity of the tested formulations was evaluated in HaCaT cells using a resazurin-based cell viability assay. HaCaT cells were seeded in 96-well plates at a density of 5 × 10^3^ cells/well and cultured overnight under standard conditions at 37 °C with 5% CO_2_. Four treatment groups were examined, including acetic acid 1%, WBEO 0.3%, buffer, and the complete micro-emulsified ear-drop formulation. Each formulation was diluted in culture medium to obtain 12.5%, 25%, 50%, and 100% of the original stock preparation.

For the short-term exposure condition, cells were exposed to each treatment for 30 min. The treatment solution was then removed, cells were gently washed with PBS, and fresh complete culture medium was added. Cells were further incubated for 24 h before viability assessment. For the prolonged exposure condition, cells were continuously exposed to the treatment formulations for 24 h. Untreated cells were used as the viability control. After treatment, resazurin reagent was added to each well and incubated for 4 h at 37 °C. Fluorescence was measured using a microplate reader at an excitation wavelength of 560 nm and an emission wavelength of 590 nm. Cell viability was calculated relative to the untreated control group, which was set as 100%.

### 2.5. Statistical Analysis

All quantitative data are presented as mean ± SEM from at least three independent experiments. Statistical analyses were performed using GraphPad Prism software. Since the collected data were continuous quantitative variables obtained from independent experimental groups, the normality of data distribution was assessed before analysis. For comparisons among multiple groups, one-way ANOVA followed by Dunnett’s or Tukey’s multiple-comparison test was used when the data followed a normal distribution. For comparisons between two groups, an unpaired Student’s *t*-test was applied. When the assumptions of normality were not met, non-parametric tests were used accordingly. Differences were considered statistically significant when *p* < 0.05. The levels of significance were indicated as follows: *p* < 0.05 (*), *p* < 0.01 (**), *p* < 0.001 (***), and *p* < 0.0001 (****).

## 3. Results

### 3.1. Quality Control of O. gratissimum Essential Oil (WBEO)

The results show that the essential oil met the requirements of the Vietnamese Pharmacopeia V for medicinal use (Table S1, Supplementary Data).

### 3.2. Validation of the Analytical Method for Eugenol Assay in the Formulation

#### 3.2.1. System Suitability

The RSD values for retention time, peak area, theoretical plates, asymmetry factors, and resolution were all less than 2%. The analytical procedure meets the system suitability requirements (Table S2, Supplementary Data).

#### 3.2.2. Specificity

The chromatograms of the test solution and the spiked test solution showed a peak with a retention time corresponding to that of eugenol in the standard solution (retention time of approximately 10.7 min). No interfering peaks were observed at the retention time of eugenol in the blank sample (Figure S1, Supplementary Data).

Upon addition of the eugenol standard to the test solution, the peak height and area of the eugenol peak increased significantly compared to the unspiked test solution.

The eugenol peak was completely resolved from other peaks present in the chromatogram. Purity analysis using the PDA detector confirmed that the eugenol peak did not contain co-eluting components. The peak purity was determined to be 99.2% (Figure S2, Supplementary Data).

#### 3.2.3. Linearity

The suitability of the regression equation was evaluated (F = 45,845.10 > F0.05 = 2.47).

The t-value for the intercept (B) was 3.92, which is less than the critical t-value (t0.05 = 6.61). Therefore, the intercept is not statistically significant.

The t-value for the slope (B0) was 225.3, which is greater than the critical t-value (t0.05 = 6.61), indicating that the slope is statistically significant.

The regression equation representing the relationship between peak area and eugenol concentration is ŷ = 53,326x. The coefficient of determination (R^2^) is 0.9999, and the linear range is 0.076–3.800 mg/mL (Figure S3, Supplementary Data).

#### 3.2.4. Limit of Detection (LOD) and Limit of Quantification (LOQ)

The LOD and LOQ for eugenol were determined to be 2.90 µg/mL and 8.77 µg/mL, respectively.

#### 3.2.5. Precision (Repeatability)

The assessment of repeatability is presented in Table S3 (Supplementary Data). The relative standard deviation (RSD) values for both retention time and peak area were less than 2%. The method for quantifying eugenol meets the requirements for repeatability.

#### 3.2.6. Intermediate Precision

RSD values of less than 2% were obtained for both the retention time and peak area of eugenol (Table S4, Supplementary Data). Therefore, acceptable intermediate precision is demonstrated by the method.

#### 3.2.7. Accuracy

Accuracy was assessed at three concentration levels: 80%, 100%, and 120% of the target concentration. The recovery percentages at all three levels were within the acceptable range of 95% to 105%. The RSD values for all recovery measurements were less than or equal to 2.0%. The analytical method demonstrates acceptable accuracy (Table S5, Supplementary Data).

### 3.3. Formulation of Eardrop Formulation Containing Micro-Emulsified WBEO

#### 3.3.1. Screening of Surfactant and Co-Surfactant

The solubilization capacity of the candidate surfactants and co-surfactants for WBEO was systematically evaluated (Table S6, Supplementary Data). The WBEO demonstrated superior miscibility and formed isotropic, monophasic systems with Tween 80, Tween 20, Cremophor RH40, ethanol, and isopropanol. In contrast, other amphiphiles exhibited phase separation or turbidity. Therefore, 03 surfactants (Tween80, Tween20, and Cremophor RH40) and 02 co-surfactants (Ethanol and isopropanol) were selected for subsequent studies.

#### 3.3.2. Selection of Surfactant-to-Co-Surfactant Ratios

The identified surfactants (Tween 80, Tween 20, and Cremophor RH40) were screened in combination with ethanol (EtOH) and isopropanol (IPA) to generate eighteen distinct amphiphilic blends (S_mix_). To evaluate the nanoemulsifying capacity of these systems, WBEO was incorporated at a constant oil-to-S_mix_ ratio of 1:1 (*w*/*w*), followed by aqueous titration. The results of this screening are summarized in Table S7 (Supplementary Data), which highlights the six candidate formulations (S03; S06; S08; S12; S13, and S17) that exhibited the highest aqueous dilution tolerance. These specific systems maintained visual isotropy and optical transparency for a 24 h post-titration period, confirming their kinetic stability and resistance to phase separation under equilibrated conditions. According to the obtained results, six S_mix_ were further tested to figure out the most appropriate.

#### 3.3.3. Construction of Pseudo-Ternary Phase Diagrams

**Figure 1:**
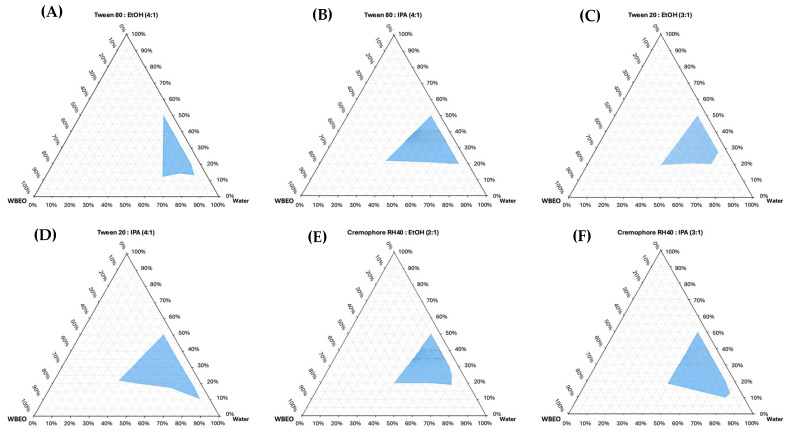
Pseudo-ternary phase diagrams of tested microemulsions for Tween 80:EtOH (**A**), Tween 80:IPA (**B**), Tween 20:EtOH (**C**), Tween 20:IPA (**D**), Cremophor RH40:EtOH (**E**), and Cremophor RH40:IPA (**F**).

A comparative topological analysis of the six pseudo-ternary phase diagrams was conducted to delineate the microemulsion regions, with the resulting quantitative metrics summarized in Figure 1.

By calculating the total area of the microemulsion region relative to the total phase diagram area, it was observed that the Tween 20 and isopropanol (4:1, *w*/*w*) system yielded the most isotropic microemulsion domain, indicating a high degree of solubilization capacity for the WBEO. This specific surfactant-to-cosurfactant architecture allowed for a significant reduction in interfacial tension while maintaining the structural integrity of the nanodroplets, even at high aqueous loading levels exceeding 50%.

In the next stage of the optimization process, two formulations with Tween 20:IPA (4:1) at a Smix concentration of less than 25% (*w*/*w*) to minimize potential otic irritation, while simultaneously maximizing the aqueous phase to a minimum of 50% (*w*/*w*) to ensure a fluid, easy-to-administer microemulsion, were tested (Table 2).

#### 3.3.4. Physicochemical Characterization of the Microemulsion

As summarized in Table 3 (for S12a) and Table 4 (for S12b), the stability profile was evaluated at five critical time points from T0 to T28, representing two distinct phases of the formulation’s lifecycle. The stability data for the two primary candidates, S12a and S12b, revealed critical differences in their electrolyte tolerance. During Phase I (T0 to T14), both formulations exhibited excellent kinetic stability, with only negligible fluctuations in mean hydrodynamic diameter and polydispersity index, well within the predefined acceptance criteria. However, a significant divergence in structural integrity was observed following the incorporation of 1% (*w*/*w*) acetic acid in Phase II. While S12a initially maintained its transparency, it failed to resist acid-induced destabilization, losing its optical isotropy and exhibiting macroscopic opacity within the first week of acidification. Consequently, S12a was eliminated from the study at T21 due to its inability to meet the required visual criteria.

In contrast, S12b demonstrated exceptional pH-resilience, maintaining its monophasic isotropic state and adhering to all physicochemical criteria through to the conclusion of the study at T28. The physicochemical characterization of the optimized microemulsion S12b over a 28-day period confirms the system’s structural integrity and short-term kinetic stability, effectively resisting the electrolyte-induced coalescence that destabilized the alternative candidates. This sustained clarity and nanostructure stability confirm the suitability of S12b as a vehicle for final otic dosage forms.

The optimized microemulsion was formulated as a concentrated amphiphilic precursor comprising 5% (*w*/*w*) WBEO, stabilized by a mixture of Smix of Tween 20 and isopropanol, as shown in the formulation below:
*Ocimum gratissimum* essential oil5%Tween 2016%Isopropanol4%Water75%

**Figure 2:**
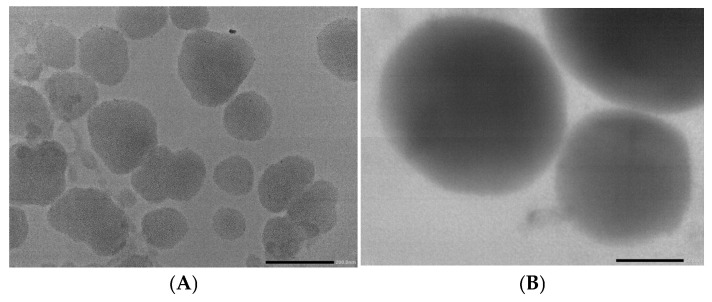
Morphology and size of the optimized microemulsion using TEM analysis. (**A**) 200 nm scale, (**B**) 50 nm scale.

The morphology and the size of the selected microemulsion were characterized using TEM analysis (Figure 2). Under TEM analysis, the optimized microemulsion demonstrates a spherical and homogenous shape with a size ranging from 50 to 150 nm. As expected, this size was slightly smaller than that measured by DLS, which can be attributed to the dehydration of droplets during sample preparation and the absence of the hydration layer in TEM measurements.

### 3.4. Evaluation of Antifungal Efficacy

To achieve the target concentration of 0.3% (*w*/*w*) for antifungal testing, the concentrated microemulsion must be diluted. The calculation is based on the mass balance equation (Table 5).

The inhibition of formulas 1, 2, and 3 against the tested fungal strains is presented in Figure S4 (Supplementary Data), and the analyzed results of the inhibition zone diameters are shown in Table 6.

Amongst the tested fungal strains, Formulas 1, 2, and 3 exhibited significant activity against *Curvularia* sp., with inhibition zone diameters of 20 mm, 20 mm, and 25 mm, respectively. Furthermore, Formula 3 demonstrated notable inhibitory activity against *Cunninghamella* sp. (27 mm), *A. terreus* (19 mm), and *Bipolaris* sp. (28 mm).

One-way ANOVA analysis, conducted to compare the inhibition zone diameters across the three formulations for each fungal strain, revealed statistically significant differences in antifungal efficacy (*p* < 0.05). This statistical significance underscores that the antifungal effect varied among the formulations, primarily influenced by the differing concentrations of acetic acid and WBEO within each formulation. This result highlights a better antifungal potential of the eardrop compared to the sole treatment with micro-emulsified WBEO or the non-microemulsified WBEO.

### 3.5. Comparison of In Vitro Antifungal Activity of the Selected Formulation and Reference Drugs

#### 3.5.1. Boric Acid 3% (*w*/*v*) Solution

**Figure 3:**
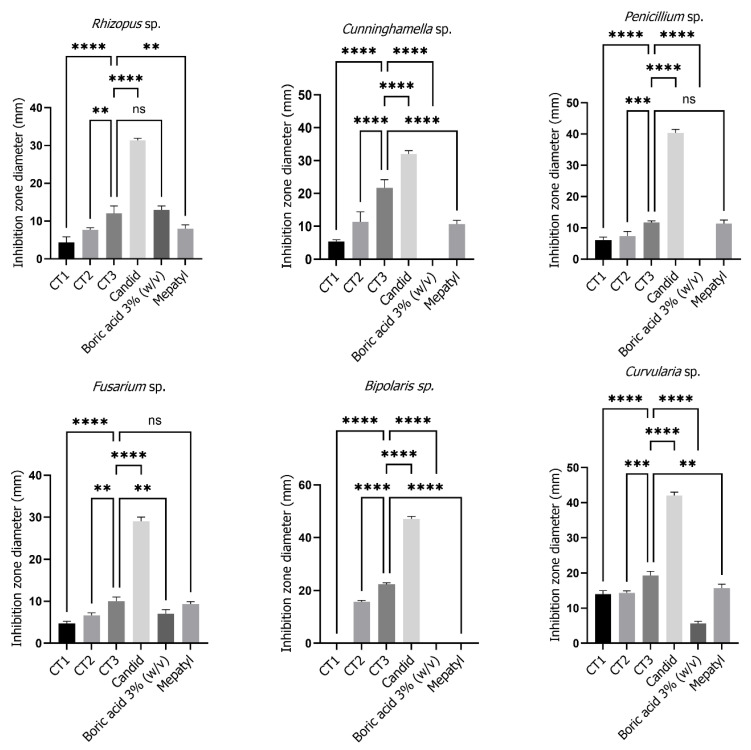

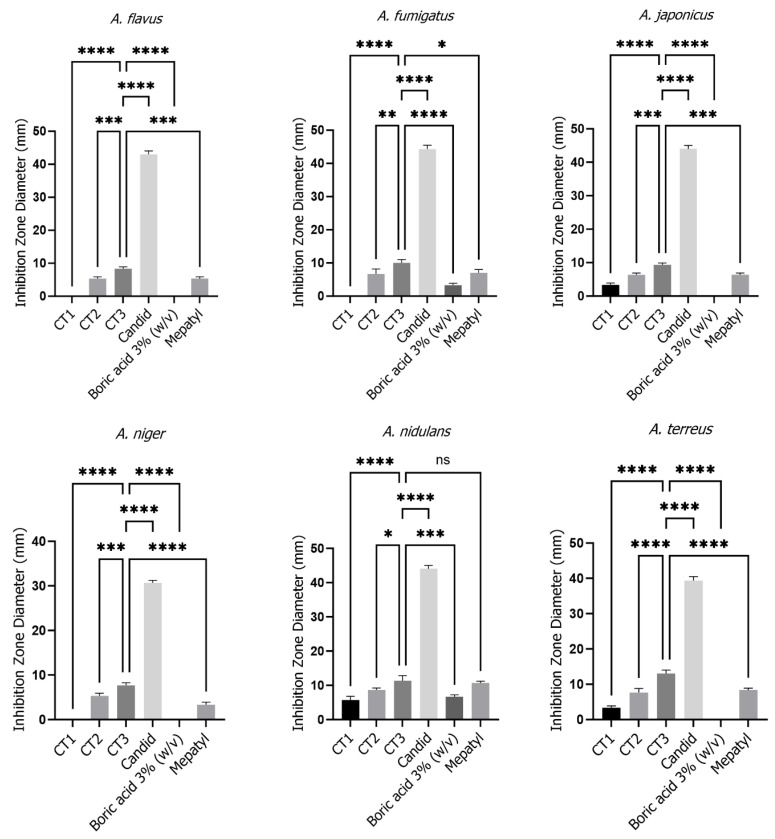
Antifungal activity on clinical isolates of the tested formulations (CT1: Formulation 1 (Control 1); CT2: Formulation 2 (Control 2); CT3: Formulation 3 (Micro-emulsified eardrop)), and reference drugs (Candid^®^, boric acid 3%, and Mepatyl^®^). (* *p* < 0.05, ** *p* < 0.01, *** *p* < 0.001, **** *p* < 0.0001, ns: non-significant).

The in vitro antifungal efficacy of the finalized formulation, comprising the microemulsified WBEO, was compared against a 3% boric acid solution (Table 7, Figure 3). The reference drug, 3% boric acid solution, exhibited no discernible antifungal activity against *A. flavus*, *A. japonicus*, *A. niger*, *A. terreus*, *Penicillium* sp., *Cunninghamella* sp., and *Bipolaris* sp. In contrast, the investigated formulation demonstrated significantly larger inhibition zone diameters against *A. fumigatus*, *A. nidulans*, *Fusarium* sp., and *Curvularia* sp. (*p* < 0.05). Notably, no statistically significant difference in activity was observed between the investigated formulation and the 3% boric acid solution against *Rhizopus* sp. (*p* = 0.4818 > 0.05).

#### 3.5.2. Acetic Acid 2% Solution (Mepatyl^®^)

The antifungal activity of the micro-emulsified eardrop was further evaluated in comparison to a reference drug containing 2% (*w*/*v*) acetic acid (Table 8, Figure 3). The results revealed that the investigated formulation exhibited significantly larger inhibition zone diameters against *A. flavus*, *A. fumigatus*, *A. japonicus*, *A. niger*, *A. terreus*, *Cunninghamella* sp., *Rhizopus* sp., *Curvularia* sp., and *Bipolaris* sp. (*p* < 0.05). Notably, the differences in inhibition were relatively small for *Curvularia* sp., *Rhizopus* sp., and *A. fumigatus*. Conversely, no statistically significant difference in antifungal activity was observed between the test formulation and Mepatyl^®^ against *A. nidulans*, *Penicillium* sp., and *Fusarium* sp. (*p* > 0.05).

#### 3.5.3. Clotrimazole 1% (Candid^®^)

The antifungal activity of the nanoformulation was also compared to a reference drug containing 1% (*w*/*v*) clotrimazole, as summarized in Table 9 and Figure 3. The results indicated that the inhibition zone diameters produced by the investigated formulation against the tested fungal strains were significantly smaller than those observed with the reference drug (*p* < 0.05).

### 3.6. Antifungal Kinetic Activity Assessment

**Figure 4:**
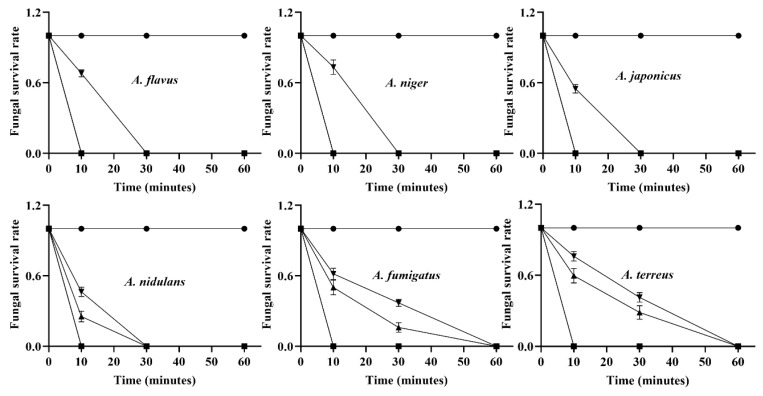

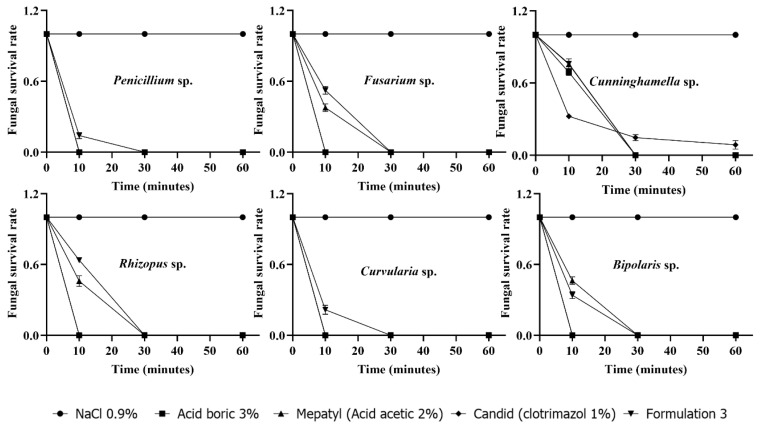
Antifungal activity kinetics of the finalized eardrop formulation (Formula 3) and reference drugs (Candid^®^, boric acid 3%, and Mepatyl^®^).

As illustrated in Figure 4, the time–kill kinetics of both the micro-emulsified eardrop and the reference drugs were investigated. A physiological saline solution (0.85% NaCl) supplemented with 0.05% Tween 80 served as the negative control throughout the experiment, ensuring that any observed antifungal effects could be attributed solely to the test agents. Viable fungal counts were determined at 10-, 30-, and 60 Min following exposure, and the proportion of surviving cells was calculated relative to the negative control.

As shown in the results, exposure to the boric acid 3% solution produced a rapid inhibitory effect. Within the first 10 min, a marked reduction in fungal viability was detected across the majority of tested strains, demonstrating the compound’s ability to interfere with fungal growth at an early stage. Prolonged exposure further enhanced this effect, as reflected by a progressive decline in the survival rate over time.

For the *Cunninghamella* sp., the onset of antifungal activity exhibited a distinct temporal profile compared to other tested organisms. Both our micro-emulsified eardrop and the boric acid 3% solution demonstrated measurable antifungal effects after 30 min of exposure, indicating a relatively rapid fungicidal action under the experimental conditions. In contrast, 1% clotrimazole required a longer contact period of 60 min to achieve a comparable reduction in viable fungal cells. These observations suggest that while all three agents are effective against *Cunninghamella* sp., their kinetics of action differ, with the optimized micro-emulsified eardrop and boric alcohol achieving fungicidal activity more rapidly than clotrimazole.

Together with the aforementioned antifungal potential, our micro-emulsified eardrop revealed dual benefits in antifungal properties, with a high efficacy and rapid activity compared to the reference drugs.

### 3.7. Quality Control of the Finalized Eardrop Formulation

All three batches of the selected micro-emulsified eardrop product containing acetic acid and micro-emulsified *O. gratissimum* essential oil met all specified criteria (Table 10).

### 3.8. Cytotoxicity of Finished Product

**Figure 5:**
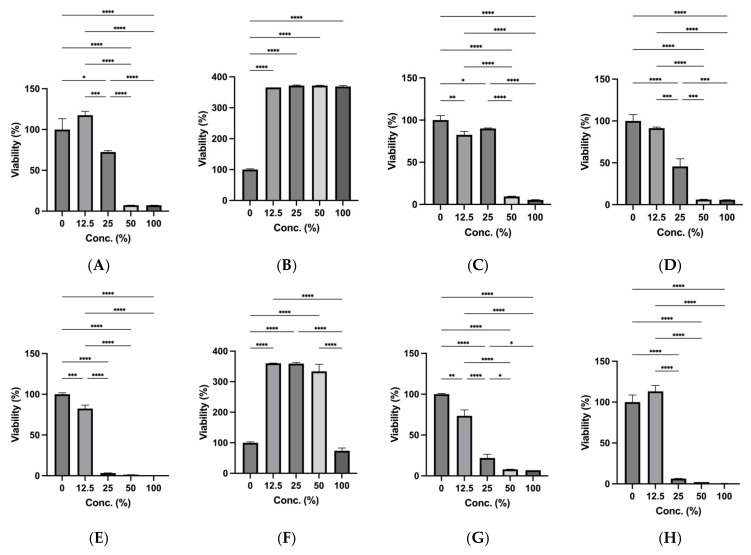
Cytotoxic effects of the tested otic formulations on HaCaT cells. Cell viability was evaluated after 30 min exposure (**A**–**D**) and 24 h exposure (**E**–**H**). (**A**,**E**) Acetic acid 1%; (**B**,**F**) essential oil 0.3%; (**C**,**G**) buffer; and (**D**,**H**) micro-emulsified ear-drop formulation. Each treatment group was tested at 12.5%, 25%, 50%, and 100% of the original stock preparation. Cell viability was expressed relative to the untreated control group. Data are presented as mean ± SD from triplicate wells. Statistical significance was determined relative to the untreated control group. * *p* < 0.05, ** *p* < 0.01, *** *p* < 0.001, **** *p* < 0.0001.

The cytotoxicity of the tested otic formulations on HaCaT cells varied according to formulation composition, concentration, and exposure duration. After 30 min exposure, acetic acid 1% reduced cell viability in a concentration-dependent manner, with relatively higher viability observed at 12.5% and 25%, whereas marked cytotoxicity was detected at 50% and 100% (Figure 5A). In contrast, the essential oil 0.3% maintained markedly elevated cell viability across all tested concentrations (Figure 5B). The buffer group also showed concentration-dependent cytotoxicity, with reduced viability at higher concentrations (Figure 5C). The complete micro-emulsified ear-drop formulation showed moderate viability at lower concentrations but substantially decreased cell viability at 50% and 100% (Figure 5D).

Following 24 h exposure, cytotoxicity became more pronounced for most tested groups. Acetic acid 1% induced severe cytotoxicity at concentrations of 25–100%, with only the lowest concentration retaining detectable viability (Figure 5E). The essential oil remained the most biocompatible treatment, maintaining high viability at 12.5–50%, although a reduction was observed at 100% after prolonged exposure (Figure 5F). Buffer exposure also reduced cell viability in a concentration-dependent manner (Figure 5G). Notably, the complete micro-emulsified ear-drop formulation caused marked cytotoxicity after 24 h exposure, particularly at concentrations of 25–100% (Figure 5H). Overall, these results suggested that the essential oil component may partially contribute to cytoprotection in the complete ear-drop formulation, particularly under short-term exposure conditions.

## 4. Discussion

This study focused on developing a micro-emulsified eardrop formulation containing acetic acid and WBEO using microemulsion as an alternative solution to synthetic agents for otomycosis treatment.

In this formulation, the surfactant/co-surfactant mixture of Tween 20 and Isopropanol at a weight ratio of 4:1 demonstrated optimal efficacy in promoting the dispersibility of the essential oil within an aqueous medium. This superior performance can be attributed to its high hydrophilic-lipophilic balance (HLB) value of 16.7, indicative of its pronounced hydrophilic characteristics. As a non-ionic surfactant, Tween 20 is a well-established excipient frequently employed in the formulation of oil-in-water emulsions and functions effectively as a solubilizer for a diverse range of active pharmaceutical ingredients, including essential oils and lipophilic vitamins. Chang et al. (2002) developed a topical suspension using Tween 20 as a surfactant [27]. Another study also revealed that Tween 20 is the optimal surfactant for essential oil solubilization [28]. The viscosity of eardrops is also a critical parameter to consider in formulation studies, as viscosity-modifying agents significantly influence the formulation’s ability to spread as a thin film and adhere effectively to the ear canal membrane. PVP K30 was selected as the viscosity-modifying agent due to its widespread availability and extensive application in pharmaceutical manufacturing. When viscosity is augmented by PVP K30, the rate of phase separation and stratification is diminished in accordance with Stokes’ Law [29].

The formulation was prepared as an oil-in-water microemulsion, a strategy employed to enhance the solubility and stability of the essential oil within the aqueous environment of the ear drop. The encapsulation of *Ocimum gratissimum* essential oil within the oil phase was a deliberate design choice intended to minimize potential volatilization of the volatile components and prevent degradation of the active compounds, thereby aiming to enhance the overall stability and extend the shelf life of the final product.

Comparative analysis of the antifungal activity against the tested fungal strains unequivocally revealed that our micro-emulsified eardrop, characterized by a composition of 1% acetic acid, 0.3% *O. gratissimum* essential oil, 0.96% of Tween 20, 0.24% of Isopropanol, and 1% of PVPK30, exhibited a relevant antifungal activity against the tested clinical fungal strains. The observed differences in inhibition zone diameters were statistically significant (ANOVA, *p* < 0.05), providing a strong indication of the formulation’s notable efficacy against the tested fungal strains, particularly *Cunninghamella* sp., which is a recognized causative agent of otomycosis. These findings are consistent with previous research by Moghadam (2010), who demonstrated the effectiveness of acetic acid solution in the treatment of otomycosis over a 3-week period [30]. Furthermore, the potent antifungal activity of acetic acid against *A. niger*, *A. flavus*, *C. albicans*, and non-*C. albicans* strains isolated directly from patients with otomycosis further substantiate the current findings and highlight the potential of acetic acid as an effective antifungal agent in otic preparations [14]. Nevertheless, the acetic acid concentration used in our micro-emulsified eardrop was diminished by a factor of two compared to these studies, diminishing the occasional skin irritation of acetic acid.

The in vitro antifungal efficacy of this newly developed micro-emulsified eardrop formulation was rigorously evaluated against three commercially available products: a 3% (*w*/*v*) boric acid solution, acetic acid 2% (*w*/*v*) solution (Mepatyl^®^), and clotrimazole 1% (*w*/*v*) (Candid^®^). Practical observations indicated that the 3% boric acid solution exhibited minimal to no efficacy when compared to our micro-emulsified eardrop formulation, particularly against strains belonging to the *Aspergillus* genus. This lack of effectiveness can potentially be attributed to the crystallization of boric acid following the evaporation of the alcohol solvent, which would impede its diffusion into the surrounding agar medium. In comparison to acetic acid 2%, the finalized eardrop formulation generally yielded larger inhibition zones across the majority of the tested fungal strains. Despite a lower concentration of acetic acid, the use of micro-emulsified *O. gratissimum* essential oil enhanced its penetration, resulting in an observed superior antifungal activity. In addition, though our micro-emulsified eardrop demonstrates a weaker in vitro antifungal activity when compared to clotrimazole 1%, the developed formulation presents a potential advantage due to the well-established broad-spectrum antimicrobial properties of both acetic acid and *O. gratissimum* essential oil, as well as a shorter required exposure time for action. Indeed, this activity is not only against fungi but also against bacteria, and other fungal groups such as yeasts and dermatophytes. For instance, Thorp et al. (1998) reported the antibacterial activity of acetic acid eardrop solution against bacteria isolated from ear discharge [31]. Furthermore, Cha Kyung Youn (2016) [32] demonstrated that acetic acid effectively inhibited the growth of *MRSA* strains isolated from ear discharge in patients with chronic otitis media. This broader spectrum of activity suggests that our finalized eardrop formulation may be beneficial in cases of mixed infections or where the specific causative agent is not yet identified [32]. In addition, as the shorter the exposure time is required for our developed product, the skin irritation effect will be significantly reduced, promoting its clinical applications both in terms of efficacy and safety.

## 5. Conclusions

In conclusion, an efficient antifungal eardrop based on microemulsion of *Ocimum gratissimum* essential oil for otomycosis treatment was successfully developed in this study. This micro-emulsified eardrop comprises 1% acetic acid, 0.3% *O. gratissimum* essential oil, 0.96% of Tween 20, 0.24% of Isopropanol, and 1% of PVPK30. The antifungal efficacy of the developed formulation was found to be superior to that of 3% (*w*/*v*) boric acid solution and 2% (*w*/*v*) acetic acid against most of the tested fungal strains. In comparison to the solution of 1% clotrimazole (*w*/*v*), the obtained micro-emulsified eardrop formulation represents a less pronounced activity but requires a shorter exposure time to be effective. All results in the current study highlight a high potential of using the micro-emulsified *O. gratissimum* essential oil and acetic acid as an efficient and safe alternative to synthetic agents for otomycosis. Further studies will assess the efficacy of the selected micro-emulsified eardrop on other fungal pathogens, especially Candida spp., as well as its ototoxicity and skin irritation.

## Figures and Tables

**Table 1:** Composition of the ear drop formulation for antifungal testing.

Ingredient	Formula 1	Formula 2	Formula 3
*Ocimum gratissimum* essential oil	0.30%	0.30%	0.30%
Surfactant/Co-surfactant	-	-	q.s.
Acetic acid	-	1.0%	1.0%
PVPK30	1%	1%	1%
DMSO	q.s.100%	q.s.100%	-
Distilled water	-	-	q.s.100%

**Table 2:** Formulation components of optimized microemulsion candidates.

No.	Smix (S:CoS)	WBEO:Smix	%WBEO	%Smix	%Water
1	S12a: Tween 20:IPA (4:1)	1:2	5.0%	10.0%	85.0%
2	S12b: Tween 20:IPA (4:1)	1:4	5.0%	20.0%	75.0%

**Table 3:** Physicochemical characterization of the optimized microemulsion S12a.

Timeline	T0	T7	T14	T21	T28
Visual assessment	Clear	Clear	Clear	Opaque	Opaque
Droplet size (nm)	160.60 ± 38.14	167.15 ± 18.20	159.97 ± 18.41	N/A	N/A
PDI	0.24 ± 0.08	0.15 ± 0.12	0.19 ± 0.11	N/A	N/A
Zeta potential (mV)	−10.3 ± 0.18	−14.2 ± 0.18	−8.7 ± 0.06	N/A	N/A
pH	5.93 ± 0.10	6.02 ± 0.10	5.91 ± 0.10	N/A	N/A
Centrifugation stability	Stable	Stable	Stable	N/A	N/A
Thermal robustness	Stable	Stable	Stable	N/A	N/A
EE%	98.39 ± 0.16%	98.09 ± 1.04%	98.85 ± 1.08%	N/A	N/A

**Table 4:** Physicochemical characterization of the optimized microemulsion S12b.

Timeline	T0	T7	T14	T21	T28
Visual assessment	Clear	Clear	Clear	Clear	Clear
Droplet size (nm)	182.50 ± 14.85	175.10 ± 22.58	165.34 ± 18.49	172.10 ± 15.86	180.30 ± 18.02
PDI	0.23 ± 0.02	0.29 ± 0.04	0.27 ± 0.03	0.24 ± 0.11	0.28 ± 0.08
Zeta potential (mV)	−9.3 ± 0.89	−9.8 ± 1.08	−8.8 ± 0.99	−10.6 ± 0.85	−10.4 ± 1.02
pH	5.99 ± 0.10	6.10 ± 0.10	5.98 ± 0.10	N/A	N/A
Centrifugation stability	Stable	Stable	Stable	Stable	Stable
Thermal robustness	Stable	Stable	Stable	Stable	Stable
EE%	98.37 ± 0.09%	97.81 ± 0.11%	98.05 ± 0.14	97.89 ± 0.16	97.93 ± 0.13

**Table 5:** Composition of the ear drop formulation.

Ingredient	Formula 1	Formula 2	Formula 3
Microemulsion			6.00 g
*Ocimum gratissimum* essential oil.			0.30 g
Tween 20			0.96 g
Isopropanol			0.24 g
Distilled water			4.50 g
*Ocimum gratissimum* essential oil	0.30 g	0.30 g	-
Acetic acid	-	1.00 g	1.00 g
PVPK30	1.00 g		
DMSO	q.s.100 g	q.s.100 g	-
Deionized water	-	-	q.s.100 g

**Table 6:** Antifungal activity of the formulations.

Fungal Strain	Inhibition Zone Diameter (mm)			ANOVA
	Formula 1	Formula 2	Formula 3	
*A. flavus*	-	11.33 ± 0.58	14.33 ± 0.58	*p* < 0.0001
*A. fumigatus*	-	12.67 ± 1.53	16.33 ± 0.58	*p* < 0.0001
*A. japonicus*	9.33 ± 0.58	12.33 ± 0.58	15.33 ± 0.58	*p* < 0.0001
*A. nidulans*	11.67 ± 1.15	14.67 ± 0.57	18.67 ± 0.58	*p* = 0.0029
*A. niger*	-	11.33 ± 0.58	13.67 ± 0.58	*p* < 0.0001
*A. terreus*	9.33 ± 0.58	13.67 ± 1.15	19.33 ± 0.58	*p* < 0.0001
*Penicillium* sp.	12.0 ± 1.0	13.33 ± 1.53	17.67 ± 0.58	*p* = 0.0018
*Fusarium* sp.	10.67 ± 0.58	12.67 ± 0.58	17.67 ± 0.58	*p* = 0.0018
*Cunninghamella* sp.	11.33 ± 0.58	17.33 ± 3.05	27.67 ± 2.52	*p* = 0.0266
*Rhizopus* sp.	10.33 ± 1.53	13.67 ± 0.58	18.0 ± 2.0	*p* = 0.0022
*Curvularia* sp.	20.0 ± 1.0	20.33 ± 0.58	25.33 ± 1.15	*p* = 0.0007
*Bipolaris* sp.	-	21.67 ± 0.58	28.33 ± 0.58	*p* < 0.0001

**Table 7:** Antifungal activity of the investigated nano-based eardrop compared to the 3% boric acid 3% solution.

Fungal Strain	Inhibition Zone Diameter (mm)		*t*-Test
	Micro-Emulsified Eardrop	Boric Acid 3%	
*A. flavus*	8.33 ± 0.58	-	*p* < 0.0001
*A. fumigatus*	16.33 ± 0.58	9.33 ± 0.58	*p* = 0.0006
*A. japonicus*	15.33 ± 0.58	-	*p* < 0.0001
*A. nidulans*	18.67 ± 0.58	12.67 ± 0.58	*p* = 0.0078
*A. niger*	13.67 ± 0.58	-	*p* < 0.0001
*A. terreus*	19.33 ± 0.58	-	*p* < 0.0001
*Penicillium* sp.	17.67 ± 0.58	-	*p* < 0.0001
*Fusarium* sp.	16.0 ± 1.0	13.0 ± 1.0	*p* = 0.0213
*Cunninghamella* sp.	27.67 ± 2.52	-	*p* < 0.0001
*Rhizopus* sp.	18.0 ± 2.0	19.0 ± 1.0	*p* = 0.4818
*Curvularia* sp.	25.33 ± 1.15	11.67 ± 0.58	*p* < 0.0001
*Bipolaris* sp.	28.33 ± 0.58	-	*p* < 0.0001

**Table 8:** Antifungal activity of the investigated formulation compared to Mepatyl^®^.

Fungal Strain	Inhibition Zone Diameter (mm)		*t*-Test
	Micro-Emulsified Eardrop	Mepatyl^®^	
*A. flavus*	8.33 ± 0.58	11.33 ± 0.58	*p* = 0.0031
*A. fumigatus*	16.33 ± 0.58	13.0 ± 1.0	*p* = 0.0213
*A. japonicus*	15.33 ± 0.58	12.33 ± 0.58	*p* = 0.0048
*A. nidulans*	18.67 ± 0.58	10.67 ± 0.58	*p* = 0.5185
*A. niger*	13.67 ± 0.58	15.33 ± 0.58	*p* = 0.0008
*A. terreus*	19.33 ± 0.58	14.33 ± 0.58	*p* = 0.0022
*Penicillium* sp.	17.67 ± 0.58	17.67 ± 1.53	*p* = 0.3486
*Fusarium* sp.	16.0 ± 1.0	15.33 ± 0.58	*p* = 0.3739
*Cunninghamella* sp.	27.67 ± 2.52	16.67 ± 1.15	*p* = 0.0034
*Rhizopus* sp.	18.0 ± 2.0	14.0 ± 1.0	*p* = 0.0363
*Curvularia* sp.	25.33 ± 1.15	21.67 ± 1.53	*p* = 0.0295
*Bipolaris* sp.	28.33 ± 0.58	-	*p* < 0.0001

**Table 9:** Antifungal activity of the micro-emulsified eardrop compared to Candid^®^.

Fungal Strain	Inhibition Zone Diameter (mm)		*t*-Test
	Micro-Emulsified Eardrop	Candid^®^	
*A. flavus*	8.33 ± 0.58	49.00 ± 1.0	*p* < 0.0001
*A. fumigatus*	16.33 ± 0.58	50.33 ± 1.15	*p* < 0.0001
*A. japonicus*	15.33 ± 0.58	50.0 ± 1.0	*p* < 0.0001
*A. nidulans*	18.67 ± 0.58	50.0 ± 1.0	*p* < 0.0001
*A. niger*	13.67 ± 0.58	36.67 ± 0.58	*p* < 0.0001
*A. terreus*	19.33 ± 0.58	45.33 ± 1.15	*p* < 0.0001
*Penicillium* sp.	17.67 ± 0.58	46.33 ± 1.15	*p* < 0.0001
*Fusarium* sp.	16.0 ± 1.0	35.0 ± 1.0	*p* < 0.0001
*Cunninghamella* sp.	27.67 ± 2.52	32.0 ± 1.0	*p* = 0.0027
*Rhizopus* sp.	18.0 ± 2.0	37.33 ± 0.58	*p* < 0.0001
*Curvularia* sp.	25.33 ± 1.15	48.0 ± 1.0	*p* < 0.0001
*Bipolaris* sp.	28.33 ± 0.58	53.0 ± 1.0	*p* < 0.0001

**Table 10:** Quality control results of the finalized eardrop formulation.

Test	Specification	Results		
		Batch1 (*n* = 6)	Batch2 (*n* = 6)	Batch3 (*n* = 6)
Appearance	Pale yellow solution	Complies	Complies	Complies
pH	2.0–4.0	2.86 ± 0.02	2.86 ± 0.03	2.88 ± 0.05
Viscosity	14.9–18.3 cP	16.6 ± 1.0	16.9 ± 1.1	17.3 ± 0.9
Microbial Limits	Total aerobic microbial count in 1 g.	Complies	Complies	Complies
	Absence of *P. aeruginosa* and *S. aureus* in 1 g.	Complies	Complies	Complies
Identification	WBEO	Complies	Complies	Complies
	Acetic acid	Complies	Complies	Complies
Assay	Acetic acid content	Complies (89.5 ± 0.2%)	Complies (89.5 ± 0.5%)	Complies (89.7 ± 0.4%)
	Eugenol content	Complies (98.5 ± 0.4%)	Complies (99.7 ± 0.4%)	Complies (95.8 ± 0.5%)

## Data Availability

The original contributions presented in this study are included in the Supplementary Materials. Further inquiries can be directed to the corresponding author.

## Supplementary Materials

The following supporting information can be downloaded at: https://www.mdpi.com/article/10.3390/pharmaceutics18060751/s1, Table S1. Quality control results for *Ocimum gratissimum* essential oil; Table S2. System suitability results; Table S3. Repeatability assessment results; Table S4. Intermediate precision assessment results; Table S5. Accuracy assessment results; Table S6. Screening for the solubility of essential oils in surfactant and co-surfactant; Table S7. Evaluation of surfactant-to-co-surfactant ratios; Figure S1. HPLC chromatograms for specificity assessment: (A) Eugenol standard; (B) Placebo; (C) Test solution; (D) Spiked test solution; Figure S2. UV spectrum (A) and purity spectrum (B) of the eugenol peak in the test solution; Figure S3. Calibration curve for Eugenol; Figure S4. Antifungal efficacy of nano-based eardrop on tested fungal strains Note: 1: Formulation 1; 2: Formulation 2; 3: Formulation 3: Nano-based eardrop; A: Mepatyl^®^; B: Boric acid 3%; C: Candid^®^.
