# Supplementary material for: A Novel Herbal Nano-Based Ear Drop with Ocimum gratissimum Essential Oil: An Alternative Strategy for Managing Otomycosis
Source: Pharmaceutics. 2026 Jun 18;18(6):751. doi: 10.3390/pharmaceutics18060751 (PMC13306609; doi:10.3390/pharmaceutics18060751)
Supplement: Supplementary file 1 [file pharmaceutics-18-00751-s001.zip › pharmaceutics-4292490-supplementary.pdf]

Supplementary materials

**Table S1.** Quality control results for *Ocimum gratissimum* essential oil

| Test | Specification | Result |
|---|---|---|
| Appearance | Clear, pale yellow liquid; characteristic aromatic odor; spicy, hot taste, causing a numbing sensation on the tongue. | Complies |
| Identification | The chromatogram of the test sample must show apeak with are tention time corresponding to that of the eugenol reference standard using HPLC- PDA. | Complies |
| Assay | Eugenol content (%) in *Ocimum gratissimum* essential oil must be greater than 60%. | Complies (75.52%) |

**Table S2.** System suitability results

| Injection | Retention time (minutes) | Peak area (mAu.min) | Theorectical plates (N) | Asymmetry factor (As) | Resolution (Rs) |
|---|---|---|---|---|---|
| 1 | 10.66 | 233808 | 40090 | 1.14 | 1.87 |
| 2 | 10.67 | 234001 | 39897 | 1.14 | 1.92 |
| 3 | 10.67 | 230398 | 40327 | 1.12 | 1.90 |
| 4 | 10.65 | 234032 | 41002 | 1.09 | 1.89 |
| 5 | 10.66 | 232983 | 39897 | 1.14 | 1.84 |
| 6 | 10.64 | 233210 | 40102 | 1.12 | 1.92 |
| Mean | 10.66 | 233072.00 | 40219.17 | 1.13 | 1.89 |
| SD | 0.0117 | 1378.6644 | 415.3194 | 0.0197 | 0.0310 |
| RSD | 0.11 | 0.49 | 1.03 | 1.76 | 1.64 |

**Table S3.** Repeatability assessment results

| No | Retention time (minutes) | Peak area (mAu.min) |
|---|---|---|
| Solution 1 | 10.8 | 295374 |
| Solution 2 | 10.7 | 294276 |
| Solution 3 | 10.7 | 293986 |
| Solution 4 | 10.8 | 289079 |

| | | |
|---|---|---|
| Solution 5 | 10.8 | 292998 |
| Solution 6 | 10.8 | 285294 |
| Mean | 10.77 | 291834.5 |
| SD | 0.0516 | 3868.91 |
| RSD | 0.48 | 1.33 |

**Table S4.** Intermediate precision assessment results

| Day | Retention time (minutes) | Peak area (mAu.min) |
|---|---|---|
| Day 1 | 10.8 | 295374 |
| Day 2 | 10.8 | 286790 |
| Day 3 | 10.7 | 290725 |
| Mean | 10.77 | 290963 |
| SD | 0.05773 | 4296.94 |
| RSD | 0.54 | 1.48 |

**Table S5.** Accuracy assessment results

| % of spiked standard | Amount of standard added (mg) | Peak area (mAu.min) | Amount of standard recoverd (mg) | Recovery (%) | Statistics |
|---|---|---|---|---|---|
| 80% | 4.00 | 496284 | 4.1830 | 104.57 | Mean = 102.94% |
| | 4.00 | 481024 | 4.0543 | 101.36 | SD = 1.61 |
| | 4.00 | 488299 | 4.1156 | 102.89 | RSD = 1.56 |
| 100% | 4.98 | 590742 | 4.9791 | 99.98 | Mean = 101.215% |
| | 4.98 | 599989 | 5.0570 | 101.55 | SD = 1.10 |
| | 4.98 | 603210 | 5.0842 | 102.09 | RSD = 1.08 |
| 120% | 6.00 | 697666 | 5.8803 | 98.01 | Mean = 99.01% |
| | 6.00 | 701269 | 5.9107 | 98.51 | SD = 1.33 |
| | 6.00 | 715615 | 6.0316 | 100.53 | RSD = 1.35 |

**Table S6.** Screening for the solubility of essential oils in surfactant and co-surfactant

| No. | Name | Role | HLB value | Result |
|---|---|---|---|---|
| 1 | Tween 80 (PEG-20 sorbitan monooleate) | Surfactant | 15.0 | Clear |
| 2 | Tween 20 (PEG-20 sorbitan monolaurate) | Surfactant | 16.7 | Clear |
| 3 | Span 20 (Sorbitan monoilaurate) | Surfactant | 8.6 | Opaque |
| 4 | Cremophor RH40 (PEG-40 hydrogenated castor oil) | Surfactant | 15.0 | Clear |
| 5 | Ethanol | Co-surfactant | - | Clear |
| 6 | Glycerol | Co-surfactant | - | Opaque |
| 7 | Propylen glycol | Co-surfactant | - | Opaque |
| 8 | Isopropanol | Co-surfactant | - | Clear |

**Table S7.** Evaluation of surfactant-to-co-surfactant ratios

| No. of $S_{mix}$ | Surfactant | Co-surfactant | Ratio S:CoS | Volume of water added |
|---|---|---|---|---|
| S01 | Tween 80 | Ethanol | 2:1 | 0,20 mL |
| S02 | Tween 80 | Ethanol | 3:1 | 0,20 mL |
| **S03** | **Tween 80** | **Ethanol** | **4:1** | **1,20 mL** |
| S04 | Tween 80 | Isopropanol | 2:1 | 0,10 mL |
| S05 | Tween 80 | Isopropanol | 3:1 | 0,10 mL |
| **S06** | **Tween 80** | **Isopropanol** | **4:1** | **1,40 mL** |
| S07 | Tween 20 | Ethanol | 2:1 | 0,10 mL |

| No. of $S_{mix}$ | Surfactant | Co-surfactant | Ratio S:CoS | Volume of water added |
|---|---|---|---|---|
| **S08** | **Tween 20** | **Ethanol** | **3:1** | **0,50 mL** |
| S09 | Tween 20 | Ethanol | 4:1 | 0,10 mL |
| S10 | Tween 20 | Isopropanol | 2:1 | 0,10 mL |
| S11 | Tween 20 | Isopropanol | 3:1 | 0,10 mL |
| **S12** | **Tween 20** | **Isopropanol** | **4:1** | **0,90 mL** |
| **S13** | **Cremophore RH40** | **Ethanol** | **2:1** | **0,50 mL** |
| S14 | Cremophore RH40 | Ethanol | 3:1 | 0,20 mL |
| S15 | Cremophore RH40 | Ethanol | 4:1 | 0,20 mL |
| S16 | Cremophore RH40 | Isopropanol | 2:1 | 0,10 mL |
| **S17** | **Cremophore RH40** | **Isopropanol** | **3:1** | **2,00 mL** |
| S18 | Cremophore RH40 | Isopropanol | 4:1 | 0,20 mL |

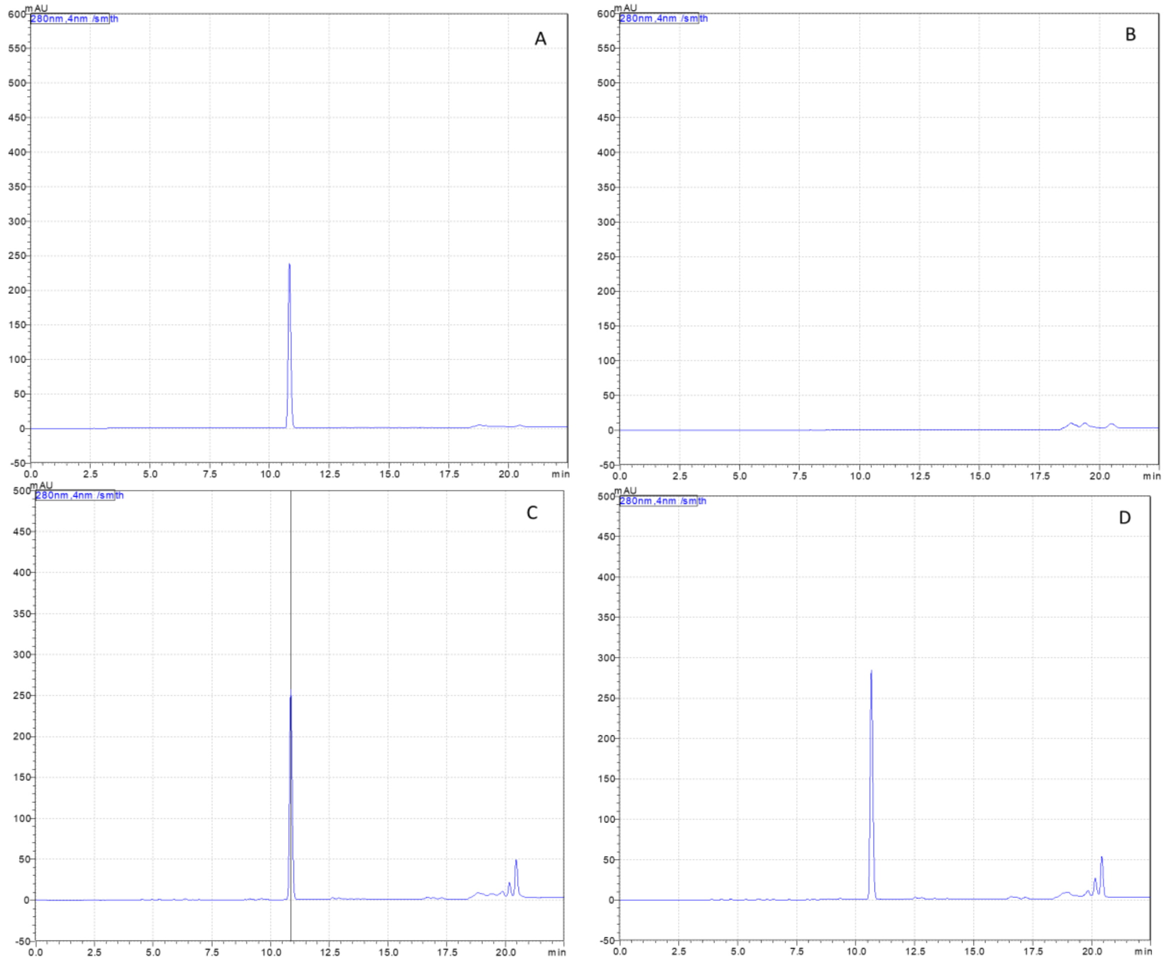


**Figure S1**. HPLC chromatograms for specificity assessment: (A) Eugenol standard; (B) Placebo; (C) Test solution; (D) Spiked test solution

The analytical procedure demonstrates sufficient specificity for eugenol.

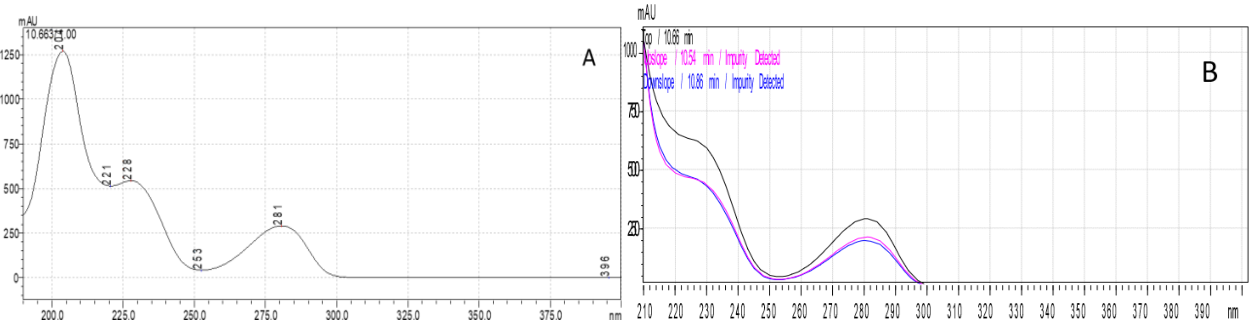


**Figure S2.** UV spectrum (A) and purity spectrum (B) of the eugenol peak in the test solution

| No | Concentration (mg/mL) | Peak area (mAU.s) |
|---|---|---|
| 1 | 0.076 | 96289 |
| 2 | 0.190 | 237958 |
| 3 | 0.380 | 450849 |
| 4 | 1.000 | 1060672 |
| 5 | 1.900 | 2005100 |
| 6 | 3.800 | 3982605 |

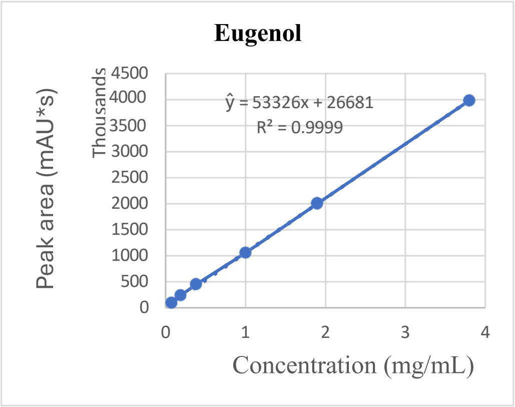


**Figure S3.** Calibration curve for Eugenol

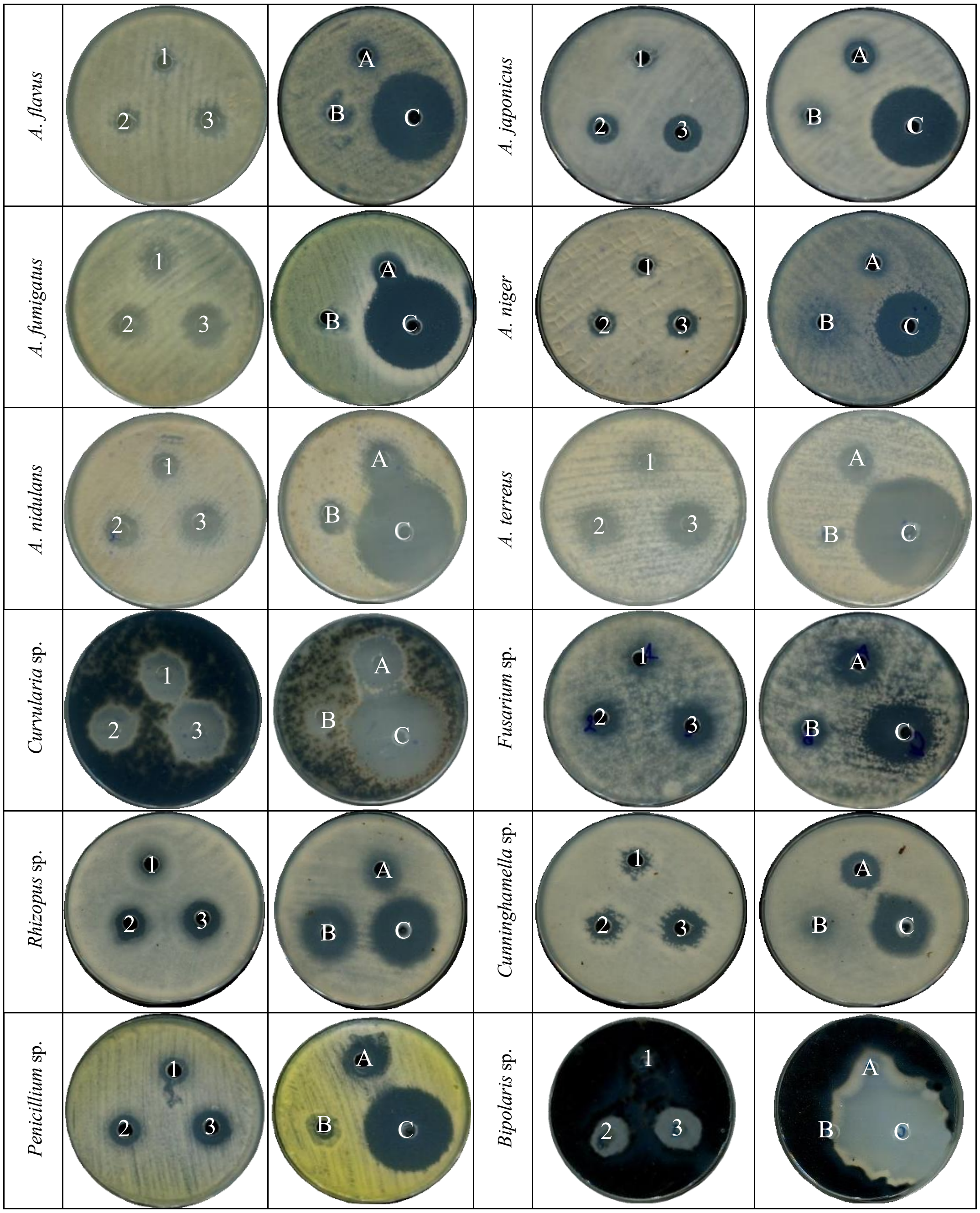


**Figure S4.** Antifungal efficacy of nano-based eardrop on tested fungal strains Note: 1: Formulation 1; 2: Formulation 2; 3: Formulation 3: Nano-based eardrop; A: Mepatyl®; B: Boric acid 3%; C: Candid®
